# Established and Emerging Methods for Protecting Linear DNA in Cell-Free Expression Systems

**DOI:** 10.3390/mps6020036

**Published:** 2023-03-30

**Authors:** Trevor J. Fochtman, Javin P. Oza

**Affiliations:** Department of Chemistry & Biochemistry, California Polytechnic State University, San Luis Obispo, CA 93407, USA

**Keywords:** cell-free protein synthesis, linear expression templates, LETs, exonuclease, recBCD, GamS, Chi, Ku, Tus-Ter, nuclease inhibition, DNA modifications

## Abstract

Cell-free protein synthesis (CFPS) is a method utilized for producing proteins without the limits of cell viability. The plug-and-play utility of CFPS is a key advantage over traditional plasmid-based expression systems and is foundational to the potential of this biotechnology. A key limitation of CFPS is the varying stability of DNA types, limiting the effectiveness of cell-free protein synthesis reactions. Researchers generally rely on plasmid DNA for its ability to support robust protein expression *in vitro*. However, the overhead required to clone, propagate, and purify plasmids reduces the potential of CFPS for rapid prototyping. While linear templates overcome the limits of plasmid DNA preparation, linear expression templates (LETs) were under-utilized due to their rapid degradation in extract based CFPS systems, limiting protein synthesis. To reach the potential of CFPS using LETs, researchers have made notable progress toward protection and stabilization of linear templates throughout the reaction. The current advancements range from modular solutions, such as supplementing nuclease inhibitors and genome engineering to produce strains lacking nuclease activity. Effective application of LET protection techniques improves expression yields of target proteins to match that of plasmid-based expression. The outcome of LET utilization in CFPS is rapid design–build–test–learn cycles to support synthetic biology applications. This review describes the various protection mechanisms for linear expression templates, methodological insights for implementation, and proposals for continued efforts that may further advance the field.

## 1. Introduction

**Figure 1:**
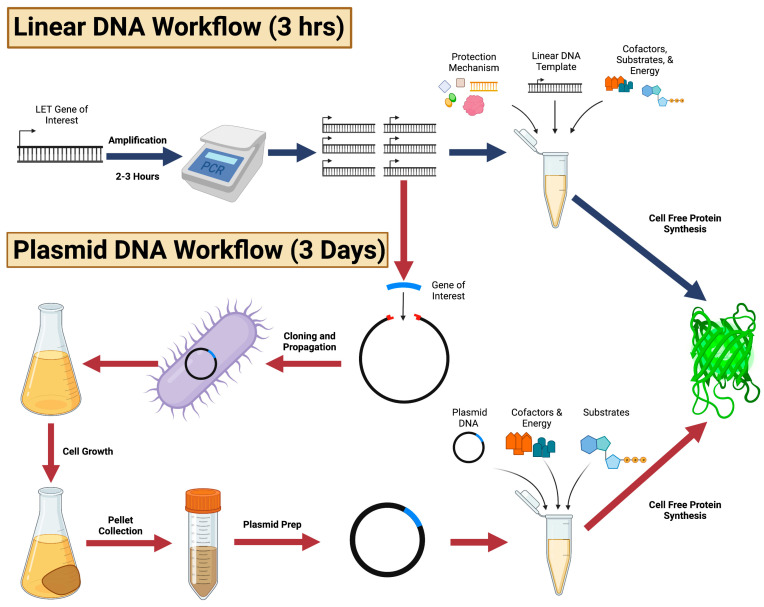
Complete workflow highlighting the major steps involved in template preparation and cell-free protein synthesis for both linear expression template (LET) DNA and plasmid DNA. Figure created with BioRender.com.

Cell-free protein synthesis (CFPS) is a reliable method for a variety of applications, ranging from on-demand protein production to synthetic circuits and artificial cells [1,2,3]. Plasmid DNA is currently the most reliable DNA template for a cell-free protein synthesis reaction due to its stability and protection from exonucleases [4]. Although effective, the process associated with the production of CFPS-compatible plasmid templates is more resource intensive and lower throughput compared to linear expression templates (LETs) [5,6]. LETs can be generated within two to four hours through PCR in contrast to the cloning and extraction of plasmid DNA workflows, which span multiple days (Figure 1) [7,8]. Despite the advantages of speed and throughput of preparation, LETs suffer from significantly lower expression yields than their plasmid counterparts [4]. Lower cell-free expression yields are the direct result of LET susceptibility to nuclease degradation [5]. To unlock the potential for LETs in cell-free applications, researchers have developed a variety of LET protection mechanisms that close the gap between linear and plasmid-based expression in cell-free systems.

The cloning, propagation, and growth processes associated with plasmid production can require a minimum of three days, while LET DNA can be produced within three hours (Figure 1). While the gene of interest can be cloned from the host genome, improved access to DNA synthesis services has obviated that workflow for most applications. The LET can be synthesized with the gene of interest and desired UTRs, T7 RNAP promoter in most cases, an optimized ribosome binding site, and a terminator. It is advisable to evaluate the LET for alternate initiation and termination sequences and undesired mRNA secondary structures. Given that linear templates can be directly utilized in CFPS, researchers are able to screen optimal LET sequences and gene variants in high throughput. In contrast, plasmid-based templates require additional preparatory steps, including cloning, propagation, and purification, which reduce the speed and throughput of screening (Figure 1). However, Plasmid DNA continues to be preferred for its stability during CFPS reactions, which results in robust expression of proteins of interest. Plasmid DNA is also preferred for experimental controls, conditions that are utilized repeatedly, and for larger scale expressions due to the large quantities per batch that can be obtained from maxi-preps.

Exonuclease V, encoded on the recBCD operon, is the main source of LET degradation even in low exonuclease extracts [5]. Some researchers have observed that linear templates completely degrade *in vitro* in under 30 min when used without protective measures, resulting in a failed reaction with incomplete template strands [9,10]. Several methods have been developed to combat the effects of Exonuclease V, as well as to eliminate it completely. Such efforts include editing the bacterial genome to modify the recBCD operon, supplementation of Exonuclease V inhibitors, and modification of the LET. These methods alone and in conjunction with one another have been shown to stabilize and protect the LET template throughout CFPS expression. Implementing the portfolio of protection mechanisms will revolutionize the design–build–test–learn cycles for synthetic biology [5]. The growing toolbox of LET protection mechanisms continues to expand the utility of cell-free systems for a variety of applications, including rapid production of proteins, support genetic circuits design *in vitro*, and improve workflows for for directed evolution efforts [4,11,12,13,14,15]. In this review, we discuss the biochemical mechanisms associated with LET protection methods. Standard working conditions, as well as an overall understanding of how to properly use these methods will also be discussed. 

## 2. Established Methods

### 2.1. Nuclease Inhibition

**Figure 2:**
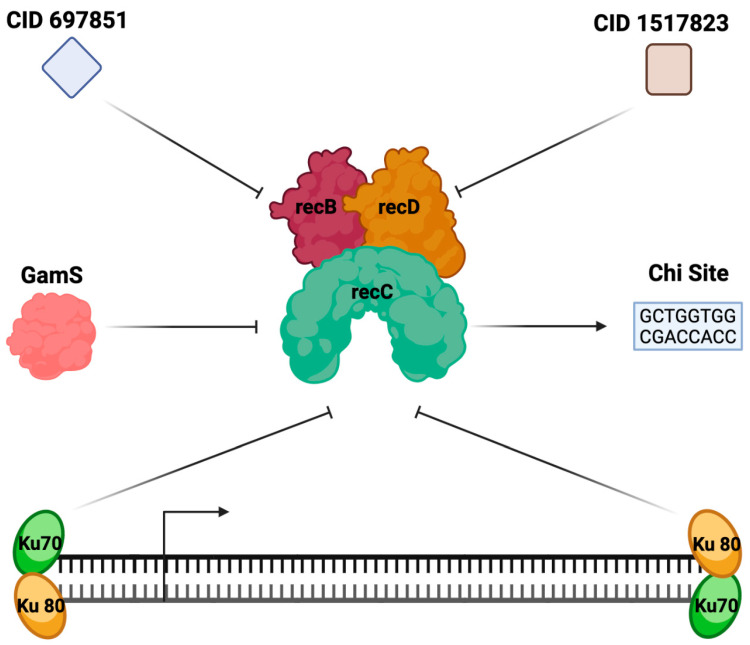
Competitive inhibition of Exonuclease V via GamS, Chi Sites, Ku proteins, CID69785, and CID 1517823. The mechanism of LET protection is displayed for each of the three inhibition strategies. Figure created with BioRender.com.

The inhibition of Exonuclease V found within the cell extract provides a modular approach to LET protection. All five inhibitors—GamS, Chi, Ku, CID 697851, and CID 1517823—can be supplemented into a cell-free system prior to or during the cell-free reaction, providing a flexible and modular option for LET protection (Figure 2). The efficacy of LET protection by each inhibitor type varies within the context of the bacterial strains used, allowing researchers to tailor protection toward their specific reaction (Table 1) [4,8].

#### 2.1.1. GamS

The first of the three inhibition mechanisms discussed in this review is a protein called GamS, one of the two gam isoforms expressed from the bacteriophage lambda. When this bacteriophage interacts with a host cell, the lambda phage integrates itself into the host genome through the lysogenic pathway [16]. The main function of the Gam protein *in vivo* is to inhibit the recBCD nuclease complex to protect bacteriophage DNA within the cell [17,18]. Acting as a steric block, GamS binds to the active site of Exonuclease V to competitively inhibit DNA binding [18]. Due to similarities in bacteriophage DNA and LET vulnerability to Exonuclease V, the implementation of GamS in a CFPS system aims to prevent Exonuclease V from binding and degrading the desired linear DNA templates [17,18].

The efficacy of GamS has been evaluated using a variety of assays, with reporter protein expression being utilized as the metric for observable LET protection. Unprotected plasmid DNA was implemented into the system as a positive control to provide a benchmark for current plasmid CFPS yields [13]. Through a series of experiments quantifying the amount of degradation of LETs over time, the working concentration of GamS was determined to be 3.5 μM in a standard *E. coli* CFPS system (Table 1) [13]. The working concentration was determined by inserting the GamS protein at various concentrations into a tube with 2 nM of linear DNA. To evaluate the effectiveness of GamS on LET stability, the protein inhibitor was tested against plasmid-based expression levels in a series of *E. coli-*based cell-free reactions. When GamS was the only protection added, the resulting protein expression reached 37.6% relative to the plasmid expression benchmark in a 105 μL, 8-h CFPS reaction [13]. When GamS supplementation was used with a LET containing 5′ and 3′ base pair extensions, an additional 20% increase in GFP expression was observed in comparison to GamS protection alone [13] Multiple reactions were conducted to optimize the maximum expression achieved with the least number of base pair additions. The most efficient number of additions for GamS was calculated to be 1000 bp at each end of the template [4]. In other efforts, the amount of RNA aptamer transcript produced from LETs has been used as a metric, rather than protein expression. In an *in vitro* transcription system monitoring the ability of inhibitors to protect LETs, GamS outperformed Chi and Ku inhibitors, yielding 700 AU of the broccoli aptamer, while the plasmid reached 2000 AU [4]. The viability of templates over time is the third measure by which LET protection is measured. Over a period of two hours, multiple samples were taken from the reaction mixture at different time points to evaluate the number of exo-resistant LET strands present in the system. With 2.58 μM GamS added to the system, nearly 60% of the LETs were fully degraded at the end of the reaction, while unprotected stranded completely degraded in 30 min [10]. In a similar experiment analyzing the degradation of PCR products protected by GamS at 117 nM, no observable destruction of LETs was noted for two hours [10]. Preserving the number of exo-resistant strands throughout the reaction mixture will allow for the reaction to proceed quickly, as well as effectively. Although GamS-mediated inhibition could not fully protect against LET degradation, the versatility of the inhibitor, as well as the ability to work in conjunction with other methods, makes GamS a viable choice when designing linear protection mechanisms.

#### 2.1.2. Chi Sites

Chi site DNA provides a linear expression template protection mechanism that is distinct from other recBCD inhibition strategies. Characterized by the nucleotide sequence of 5′-GCTGGTGG-3′, Chi sites are readily identified and bound by Exonuclease V (Table 3) [19]. Chi sites are an essential part of the homologous recombination system within *E. coli* utilized to repair DNA damage, such as double strand breaks, DNA gaps, and interstrand cross-linkages (ICLs) [20]. The DNA ends generated during intermediate steps of repair are vulnerable to Exonuclease V degradation, but they remain stabilized by the preferential binding of Exonuclease V to Chi site DNA [19]. The recBCD, an operon capable of producing Exonuclease V, is composed of three separate subunits: recB, recC, and recD. Two subunits, recB and recD, have helicase motor functions, while the recC subunit serves to identify DNA [21]. Upon binding to a Chi site, the helicase functions of the Exonuclease V are greatly reduced as the recB subunit gains motor priority over recD [21,22]. Following an interaction with a Chi site, the functionality of the exonuclease is greatly diminished, resulting in an observable reduction in LET degradation [19]. Since Chi DNA sequences occur naturally within the *E. coli* bacterial genome, implementation Chi DNA segments in *E. coli-*based extract has not been associated with adverse effects on cell-free expression or LET stability.

To evaluate how Chi sites could be adapted to CFPS applications, oligos containing 1, 4, 6, or 9 chi site repeats in various concentrations were titrated into a CFPS reaction [15]. The CFPS reaction driven by linear templates supplemented with 0.5 μM of (85 bp) dsDNA, encoding six repeats (Chi6), was observed to be effective with 2 μM of Chi9 (125 bp) (Table 1). dsDNA also proved to be a viable option [19]. The broader effective concentration range and the convenience of synthesizing 85 bp dsDNA make the Chi6 a more user-friendly option. To evaluate the effectiveness of Chi6 on protecting linear templates, deGFP expression was driven by genetic circuits containing either sigma 28 or T7 RNAP Polymerase [19]. Notably, the assay required the stabilization of a genetic circuit comprising 2 LETs, one encoding the polymerase and the other encoding the deGFP, making this a more robust method for evaluating the effectiveness of Chi-based protection. An increase in the deGFP yield of both genetic circuits was observed with the addition of Chi to a LET-based CFPS reaction [19]. The effectiveness of Chi Site DNA was also evaluated in *in vitro* transcription systems producing the broccoli aptamer [4]. In direct comparison to GamS in the aptamer-based assay, the effectiveness and expression results were nearly identical, yielding 675 AU of the broccoli aptamer upon supplementing either 3.5 μM for Gams or 0.5 μM Chi6 (85 bp) [4].

#### 2.1.3. Ku

The Ku proteins used for exonuclease inhibition are adapted from the bacterial Non-Homologous End Joining (NHEJ) pathway [20]. The NHEJ pathway is a biochemical mechanism that is essential for the full repair of double strand breaks of dsDNA within the cell [23]. While being crucial to linear DNA end protection, Ku proteins are also involved in VDJ recombination to create antigen receptor gene rearrangements [24]. For the rearrangements to occur, the Rag 1 and 2 proteins are needed to induce a site-specific double-strand break [25]. The double-strand breaks leave the ends of each strand exposed, allowing for Ku proteins to bind. Once bound, the Ku proteins block exonuclease activity to protect DNA ends from degradation during repair [25,26]. Ku proteins are comprised of two different subunits, Ku 70 and Ku 80, which bind to each strand of the dsDNA (Figure 1), preventing further degradation at the site of double strand breaks [26]. When introduced to CFPS reactions, Ku proteins are predicted to bind the ends of LETs in CFPS reactions, resulting in protection through a steric block to E xonuclease V.

The utility of Ku proteins to protect LETs in CFPS systems has been observed to be context dependent and distinct from GamS and Chi. When expressing the broccoli aptamer in *E. coli* extracts, Ku-based LET protection appeared underwhelming. Over a period of three hours, Ku protected systems only expressed 275 AU of the broccoli aptamer within an *E. coli* system, resulting in less than 50% of expression levels of LETS protected by GamS and Chi [4]. To the contrary, Ku-based protection of LETs is extremely successful in cell extracts created from *B. subtilis*, expressing over 550 AU of the broccoli aptamer. Comparatively, in *B. subtilis* extracts, GamS and Chi DNA expressed less than 300 AU of the broccoli aptamer [4]. The context dependency of Ku-based LET protection highlights the diversity and versatility of Exonuclease V inhibition, as well as the ability of users to mix and match protection strategies to suit their applications.

Given the versatility of *E. coli* for supporting robust extracts, as an extract, it allows for the expression of a variety of proteins. Because of these qualities, most expression experiments were performed in *E. coli*-based extracts [27,28,29]. In extracts prepared from other strains, differing results between the two inhibition strategies were present, opening the ability to tailor the reaction to the specific bacterial strain being used [4].

#### 2.1.4. Small Molecule Inhibitors

In addition to protein-based inhibitors, small molecule inhibitors of Exonuclease V have also been identified [30,31]. Two compounds with unique backbone structures, CID 697851 and CID 1517823, were identified from the NIH molecular libraries sample collection of over 300,000 compounds for their effectiveness in inhibiting Exonuclease V [30]. When evaluated for their capacity to improve LET-based expression in CFPS, 3.3 μM CID 697851 improved expression by two-fold, with marginal improvements to 2.5-fold at 330 μM CID 697851. CID 1517823 proved to be more effective, increasing LET-based expression by 3-fold at 5.1 uM, the lowest concentration tested (Table 1) [31]. Since the linear template controls yielded only 25% reporter protein expression compared to plasmid-based expression, the supplementation of small molecule inhibitors to a LET-driven CFPS reaction allows the user to reach 50–75% of plasmid-based expression. Given the multitude of enzymatic functions encoded by recBCD, the identification of small molecule inhibitors that do not interfere with the complex enzymatic processes within a CFPS reaction is notable. While these compounds have been demonstrated to improve LET-based expression in CFPS, they remain underutilized. A key challenge in broader adoption of their use is the poor commercial availability.

The adaptation of natural processes to inhibit Exonuclease V in CFPS demonstrates the power of synthetic biology approaches in continued platform development. As a result of efforts by the CFPS community, the availability of the inhibitors has improved [4,10]. GamS has become widely adopted and is commercially available from vendors as a companion product to CFPS kits. Ku 70/80 heterodimer is commercially available, but current vendors may be limited in some regions, and researchers continue to purify the reagent for use. Chi-site DNA is also readily accessible through DNA synthesis. Access to the small molecule inhibitors is likely the most constrained [30,31]. Given the improved availability of these reagents, the research community is poised to further characterize the combinatorial benefits of co-titrating the inhibitors to fully protect LETs in CFPS to match plasmid-based expression.

**Table 1:** List of Exonuclease V inhibitors, LET expression data, and optimal concentrations and specific mechanisms for GamS, Chi, Ku, CID 697851, CID 1517823, and Tus Ter mediated protection of LETs.

Inhibitor	Mechanism	Expression Data	Optimal Concentration	Sources
Control (Standard Linear Template)	N/A	Complete degradation of templates	N/A	[4]
GamS	Competitive Inhibitor	650 AU of the Broccoli Aptamer -Produced 5 uM deGFP	3.5–4 μM per reaction flask for a standard CFPS reaction	[4,13]
Chi Sites	Competitive DNA Binding Site	600 AU of the Broccoli Aptamer 9 μM of deGFP a 14 h reaction produced 50 μM of GFP	The most ideal concentration of the Chi Site inhibitors is 0.5 uM of the Chi6 segment (85 bp)	[4,19]
Ku	Competitive Inhibitor	Ku barely reached 200 AU Highest effectiveness in *V. Natriegens*	1–4 μM of protein is ideal	[4]
Tus-Ter	Initiation– Termination System	52,000 RFU of deGFP 40,000 RFU of mCherry	5 μM of Tus-Ter per CFPS reaction	[32]
CID 697851	Small Molecule Inhibitor	Increased yields by 200%	3.3 uM	[30,31]
CID 1517823	Small Molecule Inhibitor	Increased yields by 300%	5.1 uM	[30,31]

### 2.2. Untranslated Regions

Extending the untranslated regions (UTRs) of the linear expression template has been observed to improve protein expression, presumably by delaying the negative effects of Exonuclease V on the open reading frame encoded within the LET [33]. Since this method does not alter the general chemistry of the LET, it can be readily combined with other LET protection methods. When extending the UTRs of a LET, there are two factors that need to be considered: the length of the UTR and the sequence composition of the UTR. Determining the proper length of UTR extensions is important for balancing the gains in LET stability versus the costs of producing and maintaining an extended UTR [33]. The UTR length can be a constraint for high-throughput workflows leveraging LETs, which incur a penalty either in $/bp cost of template synthesis, or in downstream workflow complexity for augmenting the UTRs with extensions through PCR or other DNA assembly methods. When compared to the expression of a template protected by GamS alone, the addition of GamS and 5 bp at the end of each LET provided a 2.4-fold increase in expression. The increase was not linear, with expression leveling off at a six-fold increase for a 250 bp UTR at each end of the template [13].

In concert with the optimal length of the UTR, the sequence composition of the UTR further helps to maximize protection. A higher composition of guanine and cytosine nucleotides, ranging from 60–70% GC content, resulted in increased GFP expression compared to a LET dominated by adenine and thymine [33]. With a higher percentage of GC content in the UTR, the docking score for Exonuclease V is dramatically reduced, indicating a low affinity for the LET substrate. Docking scores describe the calculated affinity between an enzyme and substrate, with high docking scores indicating a higher affinity [34]. By lowering the docking score of linear templates, exonucleases are calculated to have a lower affinity toward the LET, resulting in reduced template degradation over time. Secondly, the sequence composition within the high GC content region of the UTR impacts LET stability as well. Sequences comprised of alternating Gs and Cs (GCGC) yielded levels of expression as high as the plasmid, while the sequences with an order of GGCC exhibited only 70% of plasmid expression levels [33]. While there are conflicting data in terms of the ideal length of the UTR, the high GC content of the UTR appears to be essential for LET protection.

Combinations of UTR extensions and exonuclease V inhibition methods can prevent LET degradation and increase expression over time. The most common of these pairings is the implementation of GamS in concert with UTR extensions [4,13]. When measuring the expression of GFP, 500 bp UTR and GamS were added to a LET-based system. The combined inhibition strategies yielded an average expression of 6 uM from linear templates, improving GamS-based protection from approximately 35% to nearly 50%. The plasmid yielded nearly 12 μM of protein in the same experiment. In another study evaluating transcription of the broccoli aptamer, a 1000 bp UTR extension combined with either GamS or Chi DNA yielded 500 AU [4]. In all cases, the unprotected LET templates degraded completely with little to no protein expression [4,12]. Another key observation of these studies was the plateau in LET protection as UTR length increased. Beyond a 1000 bp UTR, protection from exonucleases does appear to improve [13,33].

### 2.3. Tus-Ter Initiation/Termination System

The Tus-Ter initiation termination system is another established method of protecting LETs with reliable protein expression results. This naturally occurring mechanism within *E*. *coli* is responsible for protecting DNA at the replication forks during replication [35,36]. Made up of two parts, the Tus-Ter initiation termination system is composed of a 23-base pair ter sequence (Table 3), which is bound by the Tus protein [35,37].

To evaluate the effectiveness of the Tus-Ter system in protecting LETs, modified strands were first compared to unprotected LETs to determine stability in *E. coli* extracts. The unprotected strands completely degraded in solution in under 20 min while the Tus-Ter modified strands degraded by 80% over a period of 120 min [32]. Another major aspect of Tus-Ter incorporation is the length of the buffer region between the gene of interest and the Tus-Ter region [32]. As seen before with the addition of GC rich UTRs, more base pairs do not always result in improved LET protection. The range of buffer region lengths varied from 0–300 bp. Interestingly, a buffer region is not required at the 5′ end, while the 3′ end needs at least a 125 bp buffer [32]. When using mCherry as the reporter for CFPS, a 300 bp buffer region to the 3′ end of the LET resulted in maximum fluorescence levels for *V. natriegens-*based extracts. Notably, these LETs yielded 150% the expression of the plasmid DNA. When expressing deGFP in E. *coli extracts*, the ideal 3′ buffer region length proved to be 125 bp, yielding the same amount of protein as the plasmid at 5000 fluorescence units (RFU) (Table 1). The LET protection form Tus-Ter is noteworthy, since protein expression is rescued from nearly no expression to supporting robust quantities of protein production that rival plasmid-based expression [32]. When directly compared to the expression with GamS and control, the Tus-Ter system expressed 150% of the value of the plasmid and equal value to GamS. The degree of LET protection by GamS was significantly higher in this work compared to previous efforts, which reported only 35–50% protection compared to the plasmid control. The difference may be due to the differing context of the *E. coli* lysates and templates used, further highlighting the importance of mapping the nuances of LET protection mechanisms across CFPS systems. When expression is conducted using a *V. natriegens* extract, the GamS-protected LETs were as productive as the plasmid controls, notably, the Tus-Ter system further enhanced the expression to nearly double the yield compared to the plasmid [32]. Overall, the Tus-Ter system has displayed the most promising results in LET protection to an extent that matches plasmid-based expression.

### 2.4. Strain Engineering

Strain engineering approaches target the recBCD operon responsible for producing Exonuclease V. Two types of strain engineering efforts have been made to stabilize linear DNA templates: full deletions and subunit deletions [38]. With successful inactivation of Exonuclease V, these efforts have the potential to obviate the need for other approaches for LET protection providing a plug-and-play platform for LET-driven CFPS (Table 2).

The first of these approaches utilize full deletions of the recBCD operon segments from the *E. coli* genome prior to growth and extract preparation. Deleting the operon in its entirety has negative effects on the host, with noticeable declines in exponential growth over time [38]. Insufficient growth will create an array of problems downstream in the extract preparation phase, lowering the yield per culture. Subunit deletions, in contrary to full operon deletions, provided mixed results. In one effort, negative effects on growth were observed with a recB subunit deletion [39]. In another effort, a recB deletion was able to support a viable strain, which resulted in productive extracts that improved LET stability [5]. Deletions of recD were also compatible with a viable strain that reduced Exonuclease V activity to improve LET stability [34]. To inactivate the recBCD operon, the Lambda Red Phage recombination system, consisting of the three sequences, exo, bet, and gam, was implemented. The notable utility of the lambda phage is its activation at 42 °C and inactivation at 32 °C. While performing induction at 42 °C, the sequences will be activated through double strand breaks within the genome. The exo sequence binds to the 5′ end of the DNA, while the bet sequence binds to the 3′ end of the double-strand break. Once bound, the linear DNA cassette for recombination will be inserted into the deleted subunit [38]. The implementation of the lambda red recombination system will initiate the production of gam within the cell, inhibiting E xonuclease V activity [16,40]. Compared to the wild type, a three-fold increase in protein expression was observed over a period of 200 min. The mutant DNA displayed a maximum yield of 700 ug of protein, while the WT yielded only 220 ug of protein. In terms of protection against degradation over time, the LETs in Exonuclease V deficient extracts only degraded by 33% in 3 h, while LETs in WT extracts degraded completely within 30 min (Table 2) [39].

An aspect of cell-free reactions that is often overlooked in terms of DNA protection from exonucleases is the concentrations of the varied materials in the solution. In particular, the levels of magnesium glutamate and potassium glutamate emerged to be important for LET protection. The optimal concentration proved to be 7–8 mM magnesium glutamate and 80–140 mM potassium glutamate [41]. Combining the ideal buffer concentrations with other protection methods vastly improves protein yields compared to unprotected LETs. Combined with the recBCD mutant strain, expression levels reached 0.8 uM of sfGFP, while the plasmid reached 1.1 uM of sfGFP. Another effort including GamS inhibition yielded 0.7 μM of GFP with the control tube lacking GamS yielded 0.55 μM [41]. The expression lowered when Chi was implemented, hinting that the changing buffer concentrations could negatively affect the docking score for Chi sites affinity for recBCD. The mutant strain, therefore, continues to require additional interventions for robust LET-based expression.

**Table 2:** Growth effects and LET stability of recBCD mutant strains in comparison to the Wild Type.

Alteration to Gene	Growth Effects	Degradation Time	Expression Yields	References
Wild Type Strain (No Modificaitons)	Normal Growth	Fully degraded 100% in 3 Hrs	220 ug of protein	[33]
recBCD Deletion	Severely Limited Growth	N/A	N/A	[33]
recBCD Replacement	Normal Growth	Only degraded by 33% in 3 Hrs	700 ug of protein	[33]

## 3. Emerging Methods

### 3.1. Paranemic Crossover DNA

Paranemic Crossover (PX) is a DNA structure that can be created through the interweaving of two flanking double helices mediated by Watson and Crick base pairing to create a coaxial complex of two highly stable double helices [42,43,44]. The coaxial DNA complex is assembled by performing a melt and anneal cycle on an equimolar mixture of the DNA strands [43]. While the improvements on LET stability in cell-free systems require further validation, paranemic crossover DNA has been demonstrated to provide noticeable improvements in exonuclease resistance in comparison to other types of DNA structures. Duplex (double-stranded) DNA, double crossover (DX) DNA, and PX DNA were incubated with multiple nucleases in a series of 64-min reactions to determine the degradation times of each complex. In the reactions where the three complexes were incubated with E xonuclease V, the duplex DNA and DX DNA were degraded within 10 min. To the contrary, 90% of the PX DNA complex remained stable over the 64-min reaction [44]. The stability and exonuclease resistance that PX DNA complexes create could improve LET-based expression in cell-free systems. By initiating protection mechanisms that are extremely successful against the most prominent exonuclease, conducting genetic circuits, as well as biosensor pathways, would become more efficient. Continued efforts to evaluate paranemic crossover complexes in combination with UTR extensions and Exonuclease inhibitors will further expand the applications of LET-driven CFPS reactions.

**Table 3:** Specified sequences for DNA based protection mechanisms: Chi Sites, PX DNA, and Tus-Ter Inhibition.

Method	Type of Protection	Sequence	References
Chi Sites	Competitive Inhibitor	5′-GCTGGTGG-3′	[19]
Ter	Initiation Termination System	5′ -AATTAGTATGTTGTAACTAAAGT-3′	[32,35,36,37]
PX DNA	DNA Complex	PX1: 5′- GTGGTATCATCAATGCTATGTGTAGGCTTAGACCTGAG-3′ PX2: 5′- ACTAGGTCGCAACAGACACAATACTTGACCGAATCACT-3′ PX3: 5′- AGTGAGTCTAACAAGTCACATATCTGTGATGATCTAGT-3′ PX4: 5′- CTCAGTTCGGTGCCTAATTGTGGCATTTGCGACACCAC-3′	[42,43,44]

### 3.2. Chemical Modifications of DNA

Modifications of the DNA template provide a modular approach to stabilize DNA within a multitude of reaction environments. The types of modifications include covalent and non-covalent modifications. Covalent modifications can be introduced early in the workflow during DNA amplification using modified primers. This approach leverages modularity and flexibility of primer design. Covalent modification including DNA cross linkages created via click reactions, strand linking, and enantiomeric L-DNA, which all enhance the ability of DNA to resist exonuclease degradation [45,46,47].

Click chemistry reactions are renowned for supporting new covalent bonds between molecules in a simple and effective manner. For example, by reacting a terminal alkyne with an organic azide, a 1,2,3 triazole linkage is produced, which yields greater stability than the unreacted complex [45]. Synthesis of modified DNA has enabled the ability to “click” base pairs, as well as whole segments with multiple reactions at once. The formation of triazole-linked oligonucleotides show promise for use in CFPS due to their low levels of enzymatic degradation [45].

Another method of chemical modifications that could be implemented to increase resistance to nucleases is L-DNA. As the enantiomeric form of the standard D-DNA, L-DNA provides differential properties than its counterpart due to a complete reversal in chirality [48]. The mirrored chirality is incompatible with the stereochemistry of Exonuclease V required for binding and digesting DNA [42]. L-DNA and D-DNA were both incubated with exonucleases to test resistance and degradation times. A sample of each type of DNA was taken before and after nuclease treatment to determine which strands degraded and which did not. Following nuclease treatment, nearly 100% of the L-DNA strands remained intact, while nearly all the standard D-DNA was degraded following the end of the reaction [47]. The increased resistance to nuclease digestion provided by L-DNA improves the potential of linear templates to be used effectively in cell-free systems.

As with L-DNA, locked nucleic acids (LNA) provide direct protection against exonucleases. In the locked nucleic acid, the 2′ oxygen of the sugar is connected to the 4′ carbon via methylene bridge. The introduction of LNA into oligomers has been seen to increase overall stability and protection from nucleases [49,50]. The ease of incorporating LNAs into LETs through modified primer synthesis makes this an accessible approach to LET stabilization in CFPS.

## 4. Conclusions

The advantage of producing LETs in a fraction of the time compared to their plasmid counterparts has been limited by Exonuclease V-mediated degradation of LETs in extract-based systems [8]. Successful implementation of emerging and established LET protection mechanisms overcomes key bottlenecks in optimizing cell-free technology for a variety of applications. Competitive inhibitors, such as GamS, Ku, Chi sites, and small molecules, directly bind to Exonuclease V to reduce binding and degradation of the template DNA [4,19,40]. Competitive inhibitors are highly versatile because of their ability to function in a variety of CFPS conditions. GamS and Chi sequences provide the best protection when implemented in *E. coli*-based extract, while Ku pairs best with *V. natriegens-*based extracts [4]. As a single source of LET protection, competitive inhibitors may not be the most effective category, but they flourish when paired with other methods, such as Tus-Ter or extended UTR sequences [32]. The addition of GC rich UTRs has also been observed to improve the lifetime of the LET [33]. Exonuclease V activity can be reduced through genomic modifications with genetically engineered strains comprised of recBCD operon deletions proving to be effective. The resulting LET stability is comparable to that of competitive inhibitors and becomes more effective when implemented in conjunction with other methods [38,39]. Lastly, the Tus-Ter initiation–termination system has been present in the genome for through evolution and directly protects DNA from exonucleases. Being a two-part system, the Ter sequence adds to each end of the LET, while a tus protein encapsulates the Ter sequence. Combining protection methods within a single CFPS reaction yield expression that matches the levels of a plasmid [32]. The utility of LET stabilization methods may give way to alternate methods that enable the circularization of LETs to balance the convenience of LET preparation with the robustness of plasmid-based expression [51]. Although the established and emerging methods described in this review provide a toolbox for stabilizing linear DNA templates *in vitro*, the mRNA transcripts of these templates remain vulnerable to nucleases. Looking ahead, we propose that the stabilization of mRNA through methods such as circular RNA, may represent the next phase of development in cell-free applications [52,53,54].

Although plasmid DNA has been the gold standard of CFPS, the new LET protection mechanisms will prove to support nearly identical levels of protein synthesis. By switching to linear template-based CFPS reactions, the design–build–test cycle for synthetic biology will be greatly improved. The CFPS market is valued at over $250 million USD, with a compound annual growth rate of over 8% [55]. The growth is likely to accelerate with the recent executive order from the United States White House on advancing biotechnology and biomanufacturing, as well as due to the important role CFPS will play in the growing bioeconomy [56].

## Data Availability

Data is contained within the article.
